# Ohio College of Dental Surgery

**Published:** 1888-07

**Authors:** 


### Ohio College of Dental Surgery.

The forty-second annual commencement of the Ohio College of Dental Surgery was held at College Hall, Cincinnati, Ohio, on Wednesday, March 7, 1888, at 8 o'clock P. M.

The degree of D.D.S. was conferred on the following graduates by George W. Keely, D.D.S., President of the Board of Trustees ;

| NAME. | STATE. |
| --- | --- |
| D. S. Anderson | Ohio. |
| H. J. Bossart | Ooio. |
| E. D. Broadwell | Ohio. |
| H. W. Cleland | Ohio. |
| D. M. Clement | Ohio. |
| J. W. Cartmell | Kentucky. |
| C. B. Clark | Kentucky. |
| Mrs. Jessie Dillon | Ohio. |
| M. H. Evans | Ohio. |
| A. B. Fletcher | Ohio. |
| R. B. Foster | Minnesota. |
| W. E. Gouchenour | Wisconsin. |
| H. E. Harlan | Ohio. |
| F. Y. Herbert | Ohio. |
| J. W. Hillman | Ohio. |
| E. D. Hinkley | Ohio. |
| C. B. Hussey | Ohio. |
| I. F. Hussey | Ohio. |
| N. B. Hartwell | Indiana. |
| O. T. Hanson | Illinois. |
| J. A. Henning | Missouri |
| J. F. Hardman | Iowa. |
| C. G. Lockwood | Ohio. |
| M. A. Menges | Indiana. |
| O. S. Mills | Illinois. |
| H. H. Robinson | Ohio. |
| B. C. Reid | Indiana. |
| J. F. Rees | Kentucky. |
| A. H. Rainey | Illinois. |
| R. D. Rood | Wisconsin. |
| C. A. Schuchardt | Ohio. |
| J. B. Schunck | Ohio. |
| H. T. Smith | Ohio. |
| Mrs. Z. V. Swift | Ohio. |
| T. D. St. John | Ohio. |
| W. C. Shankland | Kentucky. |
| B. L. Shobe | Kansas. |
| W. E. Scott | Missouri. |
| T. H. Sexton | Pennsylvania. |
| J. P. Tudor | Ohio. |
| R. H. Updegraff | Kansas. |
| S. M. Ulrey | Ohio. |
| Edwin Waddel | Ohio. |
| W. W. Wallace | Ohio. |
| E. J. Ward | Indiana. |
| W. A. Windell | Canada. |

